# Camel milk-derived exosomes as novel nanocarriers for curcumin delivery in lung cancer

**DOI:** 10.17305/bb.2024.11267

**Published:** 2024-11-06

**Authors:** Mohd Saeed, Khalid Alshaghdali, Disha N Moholkar, Raghuram Kandimalla, Mohd Adnan Kausar, Farrukh Aqil

**Affiliations:** 1 Department of Biology, College of Sciences, University of Hail, Hail, Saudi Arabia; 2 Department of Clinical Laboratory Sciences, College of Applied Medical Sciences, University of Hail, Hail, Saudi Arabia; 3 Department of Pharmacology & Toxicology, University of Louisville, Louisville, KY, USA; 4 Brown Cancer Center, University of Louisville, Louisville, KY, USA; 5 Department of Biochemistry, College of Medicine, University of Hail, Hail, Saudi Arabia; 6 Department of Medicine, University of Louisville, Louisville, KY, USA

**Keywords:** Exosome, camel milk, Hail region, lung cancer, curcumin (CUR)

## Abstract

Cancer remains a leading cause of mortality, with non-small cell lung cancer (NSCLC) being a primary contributor to cancer-related deaths. Traditional treatment strategies such as chemotherapy, radiation, and hormone therapy often present challenges, including severe side effects, drug resistance, and toxicity. Recent advancements in nanotechnology aim to enhance the effectiveness of cancer therapies by targeting drugs selectively and specifically to tumor cells. Among these innovations, exosomes, or small extracellular vesicles (sEVs), have emerged as promising carriers for drug delivery due to their natural origin and ability to encapsulate both small molecules and biologics. This study explores the use of exosomes derived from camel milk in Hail, Saudi Arabia, as a vehicle for delivering curcumin (CUR), a polyphenol with known chemopreventive properties but limited bioavailability. Camel milk was processed to isolate exosomes through differential centrifugation, followed by characterization using dynamic light scattering, zeta potential measurements, and Western blot analysis to confirm exosomal markers. The encapsulation of CUR into camel milk-derived exosomes demonstrated a 20% loading efficiency as analyzed by UPLC. In vitro antiproliferative assays revealed that the exosomal formulation of CUR (ExoCUR) significantly enhanced cytotoxicity against drug-sensitive (A549) and taxol-resistant (A549TR) lung cancer cells compared to free CUR. Molecular docking studies and molecular dynamics simulations indicated that CUR has a strong binding affinity for the epidermal growth factor receptor (EGFR), comparable to the established drug gefitinib. Furthermore, CUR effectively downregulated EGFR and STAT3 expression in lung cancer cells, suggesting its potential to disrupt key signaling pathways involved in tumor progression. Our findings highlight the potential of camel milk-derived exosomes as an effective and biocompatible delivery system for CUR, offering a promising strategy to overcome the limitations of current cancer therapies and enhance the therapeutic efficacy of chemopreventive agents.

## Introduction

Cancer is the second leading cause of mortality in the United States, with an estimated 2 million new cases and 611,720 fatalities projected for 2024 [1]. Lung cancer, especially non-small cell lung cancer (NSCLC), accounts for the majority of cancer-related deaths. Various treatment methods—including hormone therapy, radiation, chemotherapy, and surgery—have been employed in cancer treatment. However, these therapies often lead to severe health complications, primarily due to the toxicity associated with intravenous bolus dosages and the development of drug resistance.

Recent advancements in nanotechnology aim to enhance the efficacy of chemopreventive agents and therapeutic medications. Over the past few decades, researchers have explored various polymers, hydrogels, lipids, inorganic materials, and other biological carriers to target chemotherapeutics specifically to tumor cells [2]. Despite substantial efforts by the scientific community, clinical trials for these nanomedicines have shown poor success rates due to toxicity concerns, and most of these formulations have not yet been used in clinical settings. The search for innovative drug delivery systems that can achieve cost-effective and efficient treatments continues.

In living systems, cells primarily interact through extracellular vesicles (EVs), such as apoptotic bodies, shedding microvesicles, and exosomes [3]. Exosomes are naturally occurring nanovesicles ranging in size from 30 to 150 nm. They are formed when the cell membrane invaginates, creating small vesicles within a multivesicular body (MVB). When these MVBs fuse with the plasma membrane, the exosomes are released into the extracellular space [4]. Exosomes can be obtained from various sources, including cell cultures [5], plants [6], and bovine milk [7], and exosome mimetics [8], have been used for various biological applications. Exosomes have a lipid bilayer structure and contain numerous membrane-bound and internal proteins, integrins, immunoglobulins, and cell-specific surface peptides. They also carry a diverse array of RNA and DNA within their core [9, 10]. Due to their unique characteristics and nanoscale dimensions, exosomes have the potential to serve as carriers for both small and large molecules, enabling targeted delivery to tumor sites.

Exosomes have garnered increased attention with the discovery of their presence in antigen-presenting cells and their ability to activate immunological responses, transport both small and large substances, and facilitate intercellular communication. Exosomes offer a unique opportunity for drug delivery due to their nanometric size and natural origin. However, a significant challenge remains in obtaining exosomes in large quantities.

Recent research indicates that camel milk can be an effective source for isolating exosomes [6, 11]. By leveraging exosome technology, the efficacy of various anti-cancer medications can be enhanced, leading to improved therapeutic outcomes. Several studies have shown that exosomes isolated from food sources can have substantial effects on biological processes [12, 13]. Milk, in particular, is an abundant source of exosomes, which have been utilized for various biological applications [14, 15]. Milk-derived exosomes are non-toxic, non-immunogenic, and exhibit cross-species biocompatibility. Exosomes can carry both hydrophilic and lipophilic drugs to targeted sites, and their surfaces can be modified to achieve tissue-specific biodistribution [16, 17]. Previous studies have used milk-derived exosomes as carriers for chemotherapeutic agents, natural compounds, and siRNAs [18–20]. Through systematic efforts, our laboratory has identified camel milk as a biocompatible and economically viable resource for the large-scale extraction of exosomes.

Plant-derived compounds possess a wide range of biological activities, including cancer chemopreventive and therapeutic properties [21]. However, many plant compounds suffer from low bioavailability [21]. Unlike in animal studies, the relatively small amounts of natural substances consumed in the human diet, along with phase-II metabolism, limit the effectiveness of potent polyphenols in humans. Curcumin (CUR), a polyphenol known for its chemopreventive effects, is commonly used as a supplement for treating various inflammatory diseases [22, 23]. However, CUR has low bioavailability and limited ability to reach systemic organs in its unbound, non-conjugated form [24, 25]. Strategies to prevent the rapid metabolism of bioactive compounds like CUR are therefore appealing, as they may enhance CUR’s effectiveness in cancer treatment [24].

This study describes the process of encapsulating CUR in exosomes isolated from camel milk. The objective was to assess the ability of exosomal CUR (ExoCUR) to target cancer cells directly, bypassing liver metabolism. We also evaluated the antiproliferative efficacy of ExoCUR and compared it to that of free CUR in human lung cancer cells.

## Materials and methods

### Exosome isolation and characterization

Organic camel milk was collected from a small camel farm in the Hail region of Saudi Arabia, including colostrum samples collected within 1–2 days post-birth. Exosomes were isolated from the milk using differential centrifugation, following the same protocol used for bovine milk [7]. Briefly, the milk was first centrifuged at 13,000 rpm for 30 min at 4 ^∘^C in 250 mL centrifuge bottles (Nalgene, Thermo Fisher Scientific, Holtsville, NY, USA) to remove fat globules, casein aggregates, and other unwanted particles. The resulting whey was then transferred to 70 mL polycarbonate tubes and centrifuged at 100,000 × *g* for 60 min at 4 ^∘^C to eliminate large particles and microvesicles. Approximately 50 mL of the remaining supernatant was carefully collected from the upper layer and subjected to ultracentrifugation at 135,000 rpm for 90 min at 4 ^∘^C. The exosome pellet was then washed with PBS, resuspended in PBS to create a homogeneous solution, and sterilized by filtration through a 0.22 µm filter. Exosomes were stored in aliquots at −80 ^∘^C until further experimentation.

### Protein determination

Following the manufacturer’s guidelines, a small portion of the milk exosome preparation was used to measure protein concentration using a BCA kit (Thermo Scientific, IL, USA). Protein content was compared across three exosome preparations, each typically diluted 10-fold. 

### Characterization of camel milk exosomes (Exo)

Exosomes were characterized based on physical parameters, such as size, charge, and zeta potential. Size distribution was measured using a Zetasizer (Malvern Instruments Ltd., Worcestershire, UK) [26], with each sample analyzed in triplicate. For size and zeta potential measurements, exosomes were diluted to a concentration of 1 mg/mL in 0.1X PBS and analyzed for zeta potential (electronegativity) and size distribution, following the manufacturer’s instructions. Atomic force microscopy (AFM) was also conducted to assess the size and morphology of the exosomes.

### Western blot analysis

**Figure S1.**
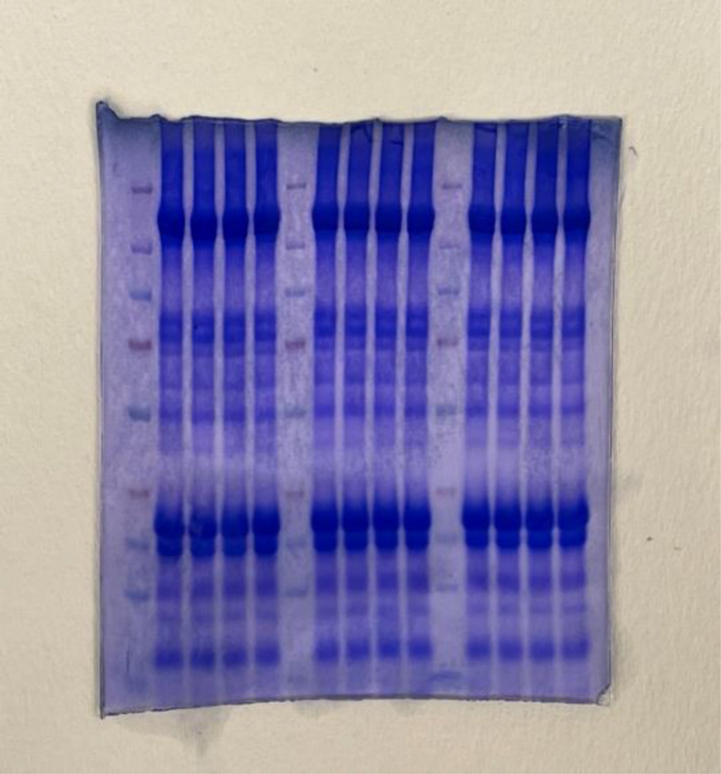
Gel staining with Coomassie brilliant blue dye showing equal loading of the proteins and serve as loading control for Figure 1C.

Exosomes were subjected to Western blotting, as previously described [27], to identify exosomal surface protein markers. Alix, CD63, and CD81 (Thermo Fisher Scientific and Santa Cruz Biotechnologies) were used as probes for detection. The experiment utilized appropriate secondary antibodies and an enhanced chemiluminescence reagent (Thermo Scientific, Waltham, MA, USA) to visualize the bands. Equal amounts of exosomal protein were loaded, as confirmed by gel staining with Coomassie Brilliant Blue dye (Figure S1).

### Drug encapsulation

CUR was dissolved in a 50:50 mixture of ethanol and acetonitrile, then combined with exosomes. The mixture was incubated for 15 min, after which free drug was removed via low-speed centrifugation. Drug-loaded exosomes were then isolated using ultracentrifugation (135 K for 2 h). The resulting CUR-loaded exosomes (Exo-CUR) pellet was resuspended in PBS.

### Drug load determination

To determine the drug loading capacity, 50 µL of Exo-CUR was mixed with 1 mL of a 50/50 acetonitrile/ethanol (v/v) solution. The samples were then centrifuged to separate the precipitated exosomal proteins, and the drug concentration in the supernatant was measured via high-performance liquid chromatography (HPLC) following the previously documented protocol [28]. The protein content of the pellets was quantified using the bicinchoninic acid assay.

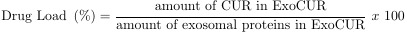


### Cell culture

Human lung cancer A549 cells were obtained from the American Type Culture Collection (Manassas, VA, USA), while taxol-resistant A549TR cells were provided by Dr. Bruce Zetter from Children’s Hospital Boston, Harvard Medical School (Boston, MA, USA). Both A549 and A549TR cells were cultured in RPMI medium (Gibco, Waltham, MA, USA) supplemented with 10% fetal bovine serum (FBS) and antibiotics (penicillin/streptomycin), maintained at 37 ^∘^C in a 5% carbon dioxide (CO_2_) atmosphere.

### Antiproliferative activity

The antiproliferative effects of CUR and Exo-CUR against lung cancer cells (A549 and A549TR) were evaluated using an MTT assay, as previously described [27]. Cells were seeded in a 96-well plate at a density of 3000 cells per well and allowed to adhere overnight. The cells were then exposed to the drug—either in its free form or encapsulated in exosomes—at various concentrations. After 72 h of treatment, the media was replaced with fresh media containing MTT (0.5 mg/mL) and incubated for 2 h. The media was subsequently removed, and the resulting formazan crystals were dissolved in dimethyl sulfoxide (DMSO). Optical density was measured at 570 nm using a spectrophotometer to assess cell viability.

### In silico studies

#### Molecular docking

To retrieve the two-dimensional structures of the target compounds—CUR (CID: 969516) and gefitinib (CID: 123631)—the PubChem database was utilized. These molecules were then converted into three-dimensional structures using Discovery Studio. The target protein, epidermal growth factor receptor (EGFR) (PDB ID: 1m17), was retrieved from the Protein Data Bank. To prepare the protein for docking, we removed heteroatoms and water molecules and performed energy minimization using the CHARMm force field in Chimera software. Following preparation, the ligands (CUR and gefitinib) and target protein (EGFR) were docked using AutoDock 4.2 under standard conditions. The best docking poses were selected and saved in PDF format for subsequent molecular dynamics (MD) analysis.

#### MD simulation analysis

Gromacs version 2020.4 was used to perform MD simulations of the 01_EGFR_Gefitinib and 02_EGFR_Curcumin complexes. Each protein–ligand complex was solvated in a truncated octahedral box with TIP3P water molecules, maintaining a minimum distance of 10 Å between the protein systems and the edges of the simulation box to ensure proper minimum image convention. The systems were neutralized with ions: 35 K^+^ and 37 Cl^−^ for the 01_EGFR_Gefitinib complex, and 35 K^+^ and 36 Cl^−^ for the 02_EGFR_Curcumin complex. Protonation states for His, Lys, Arg, Asp, and Glu residues were assigned at pH 7.4 using CHARMM-GUI, and verified by visual inspection before implementation. The 01_EGFR_Gefitinib complex contained a total of 42,580 atoms, while the 02_EGFR_Curcumin complex contained 42,620 atoms. All input files were generated using the CHARMM-GUI web server [29, 30].

Each system underwent energy minimization for 5000 steps using the steepest descent technique, achieving convergence under a force threshold of 1000 kJ/mol/nm to resolve steric clashes. Following minimization, each system was equilibrated separately. Equilibration in the NVT ensemble (constant moles, volume, and temperature) was performed for 500 ps, followed by equilibration in the NPT ensemble (constant moles, pressure, and temperature) for 10,000 ps. The NVT equilibration used a 0.1 fs time step and a temperature of 310.15 K, while the NPT equilibration also maintained a temperature of 310.15 K, with pressure set to 1 atm [31].

During the NPT equilibration and production run, weak coupling velocity re-scaling (modified Berendsen thermostat) and the Parrinello–Rahman algorithm were used, with relaxation times of τ *T* ═ 0.1 ps and τ *P* ═ 2.0 ps. For the production run, a time step of 2 fs was applied, and the LINear Constraint Solver (LINCS) algorithm was employed to maintain optimal bond lengths for hydrogen atoms. Short-range non-bonded interactions were computed with a 12 Å cutoff using the Verlet algorithm, while long-range electrostatics were calculated with the Particle Mesh Ewald (PME) method. Periodic Boundary Conditions (PBC) were applied in all directions (*x*, *y*, and *z*).

Each complex was subjected to a 120 ns production run, with trajectory and energy data recorded every 20 ps. GROMACS 2020.4 with the CHARMM36m force field was used for all MD simulations [32, 33]. Data plots were generated using the GRaphing, Advanced Computation, and Exploration (Grace) software. Finally, the molecular mechanics Poisson–Boltzmann surface area (molecular mechanics generalized born and surface area [MMGBSA]) method was used to calculate protein–ligand binding energy every 1 ns for both systems [34].

### Effects of CUR on EGFR and downstream signaling expression by Western blot

Human lung cancer A549 cells were treated with various concentrations of CUR for 48 h. Cell lysates were prepared using RIPA buffer supplemented with a protease inhibitor cocktail (Thermo Fisher Scientific, Waltham, MA, USA). Protein concentrations in the lysates were determined using the BCA Protein Assay Kit (Pierce, Rockford, IL, USA). Equal amounts of protein were then separated by SDS-PAGE, and Western blot analysis was performed as described previously. Briefly, after transferring the proteins to a PVDF membrane, specific antibodies were used to probe the membrane for target proteins (EGFR, STAT3, RAS, MEK, ERK, AKT, PI3K). Protein levels were visualized with ECL detection reagents (Thermo Scientific, Rockford, IL, USA). The densities of the protein bands were quantified relative to the internal loading control (β-actin) using ImageJ software (NIH, Bethesda, MD, USA).

## Results

### Characterization of camel milk-derived exosomes

**Figure 1.**
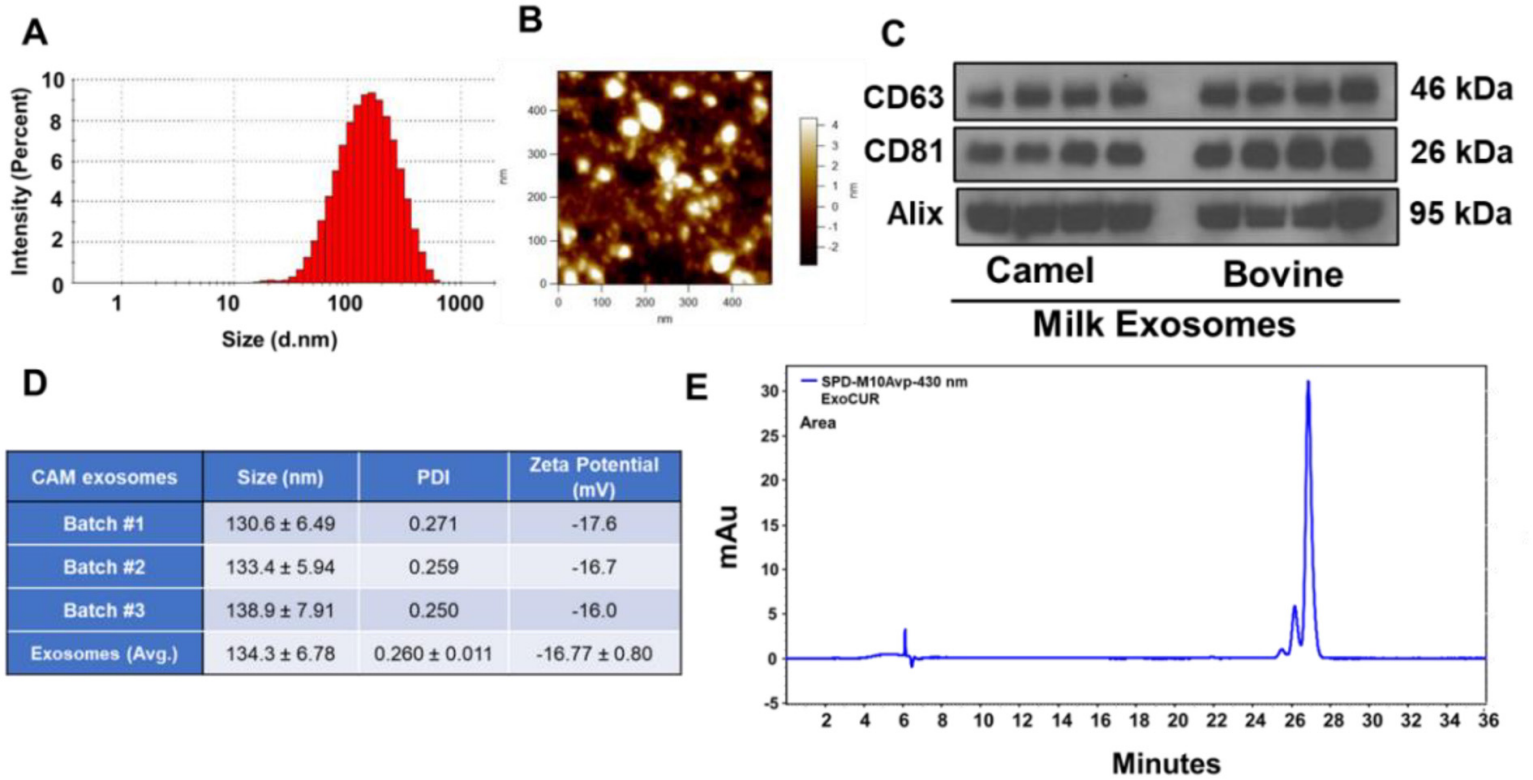
**Characterization of camel milk-derived exosomes.** Size, PDI, and zeta potential of exosomes were analyzed by Zetasizer and AFM (A, B, and D). Exosomal surface markers (CD63, CD81, and Alix) were analyzed by Western blot from camel milk exosomes and compared with bovine milk exosomes (C). Curcumin was extract by solvent extraction from Exo-CUR and analyzed by HPLC. Chromatogram shows the peaks corresponding to curcuminoids III, II, and I (E). Abbreviations: PDI: Polydispersity index; Exo-CUR: Exosomal curcumin; HPLC: High-performance liquid chromatography.

**Figure S2.**
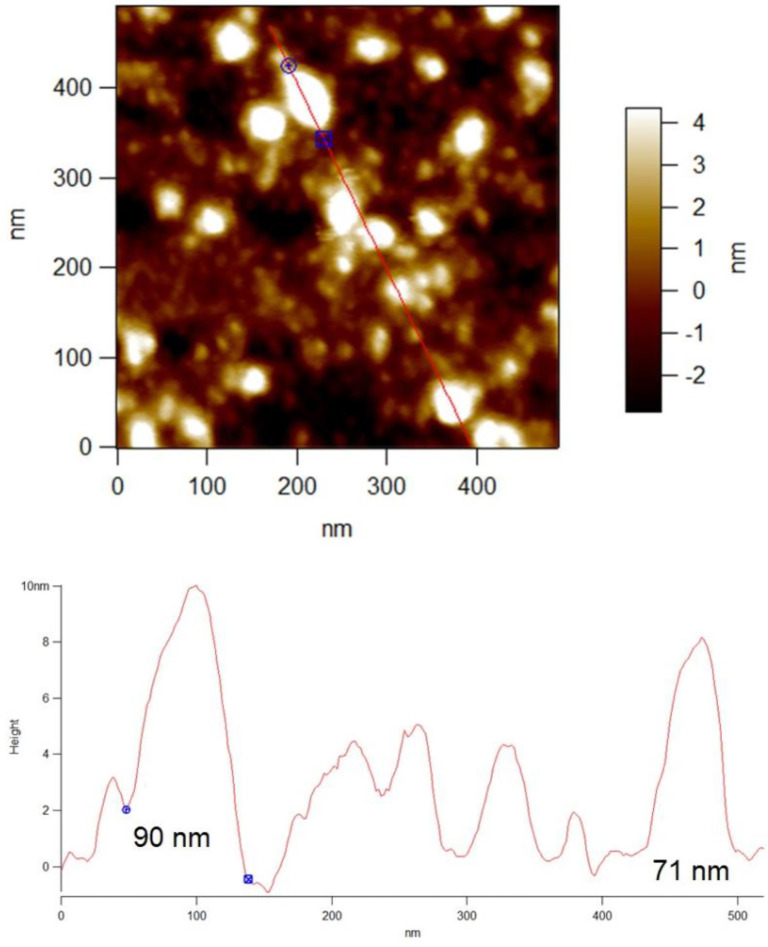
**AFM images of camel milk exosomes.** Exosomes were diluted and loaded on clean silicon wafers and air dried for 30 min. AFM images were captured using asylum research, oxford instrument with a fixed force (<1 nN) with a scanning rate if 1 Hz. Abbreviation: AFM: Atomic force microscopy.

To evaluate the therapeutic potential of exosomes, it is essential to understand their stability, homogeneity, and biophysical characteristics. Dynamic light scattering (DLS) was utilized to investigate three key parameters: size, polydispersity index (PDI), and zeta potential. The results, depicted in Figure 1, provide valuable insights into the characteristics of camel milk-derived exosomes (Exo). Exosomes were isolated in multiple batches, and each batch was assessed for size, zeta potential, and PDI (Figure 1A, 1B, and 1D). The exosomes displayed a size distribution of 134.3 nm and a zeta potential of −16 mV. This negative charge effectively inhibits aggregation and ensures stability in suspension. The exosomes had a notably low PDI of 0.26 ± 0.01, indicating a homogeneous and consistent population with potentially similar functional characteristics (Figure 1D). The size of the exosomes was further confirmed through AFM analysis (Figure 1B and Figure S2).

To verify that the isolated particles were indeed exosomes across different batches of Exo samples, we conducted Western blot analysis to detect CD63, CD81, and Alix, essential exosomal membrane markers. These markers were compared with well-established bovine milk exosomes. CD63, CD81, and Alix play key roles in the endosomal sorting of exosomal cargo, and their presence in camel milk samples suggests active exosome biogenesis via the endosomal pathway. As expected, distinct bands for CD63, CD81, and Alix were observed at the predicted molecular weights in Exo samples from two separate batches, showing a pattern similar to that in bovine milk exosomes (Figure 1C). The high levels of CD63, CD81, and Alix in the Exo samples confirm the successful isolation of exosomes from camel milk. Equal loading was confirmed by Coomassie staining (Figure S1).

### Drug loading and entrapment efficiency

To assess the potential of exosomes as nanocarriers for enhancing CUR delivery, we used HPLC to determine the loading efficiency of this bioactive compound into exosomes. HPLC provided a precise quantitative assessment of CUR encapsulation within our exosomal preparations. The exosomes were isolated and characterized, yielding a protein concentration of 2.3 mg/mL. The loading process involved incubating exosomes with an initial CUR concentration of 0.433 mg/mL. This carefully chosen ratio was intended to optimize encapsulation while maintaining exosomal integrity. Subsequent HPLC analysis revealed a loading efficiency of 18.8%, indicating successful encapsulation of CUR within the exosomes (Figure 1E). These results suggest that exosomes are a viable option for delivering CUR, potentially enhancing its bioavailability and therapeutic efficacy.

### Antiproliferative effects of CUR and Exo-CUR

**Figure 2.**
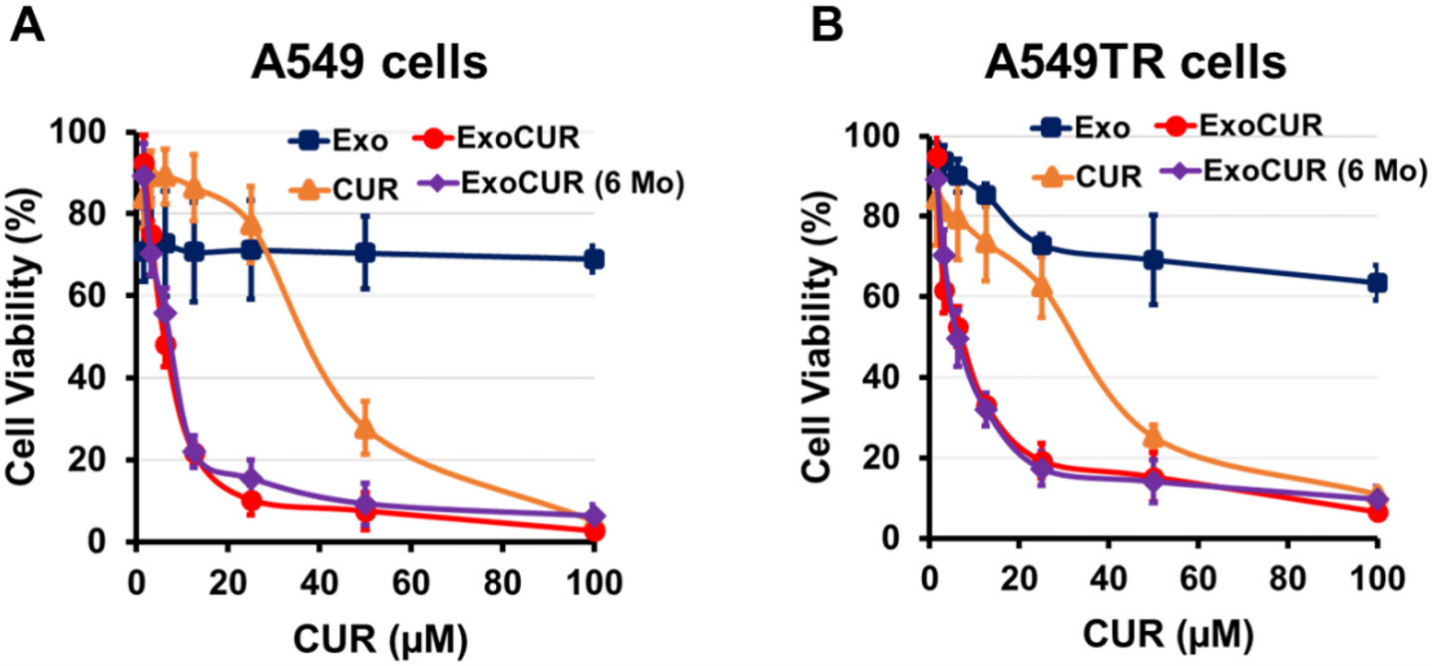
**Effect of CUR and its Exo-CUR on the cell viability drug sensitive A549 (A) and drug resistant (A549TR) (B) lung cancer cells.** Lung cancer cells were treated with varying concentrations of Exo, CUR, Exo-CUR, and Exo-CUR after 6 months storage at −80∘ C (Exo-CUR [6 Mo]). After 72 h, cell viability was determined by MTT assay. In Exo and Exo-CUR formulations, the exosomes concentrations were 0–100 µg/mL. Data represent mean (± SD) of three independent experiments. Abbreviations: Exo: Exosomes; CUR: Curcumin; Exo-CUR: Exosomal curcumin.

The results shown in Figure 2A and 2B demonstrate a striking enhancement in the cytotoxic efficacy of CUR when delivered via exosomes. While exosomes and CUR alone displayed modest antiproliferative effects, the combination in the exosome-loaded form exhibited significantly greater efficacy against the tested cancer cells. At all tested concentrations, Exo-CUR showed markedly higher cytotoxicity compared to either treatment alone. At a dose of 50 µM, Exo-CUR achieved 12% cell viability, compared to 24% for CUR alone at the same dose, while exosomes alone had minimal effect. This substantial improvement suggests that exosomes act as efficient delivery vehicles, facilitating CUR uptake into the cells and potentially protecting it from degradation. The efficacy of Exo-CUR was further evaluated in A549TR lung cancer cells, where a similar pattern was observed (Figure 2B). Exo-CUR effectively suppressed cell viability, even at very low doses, inhibiting 80% of cells, compared to free CUR, which left 20% of cells viable. Notably, exosomal formulations decreased the IC50 values by 6- to 8-fold in both drug-sensitive (A549) and drug-resistant (A549TR) cell lines (Figure 2).

### Molecular docking analysis

**Figure 3.**
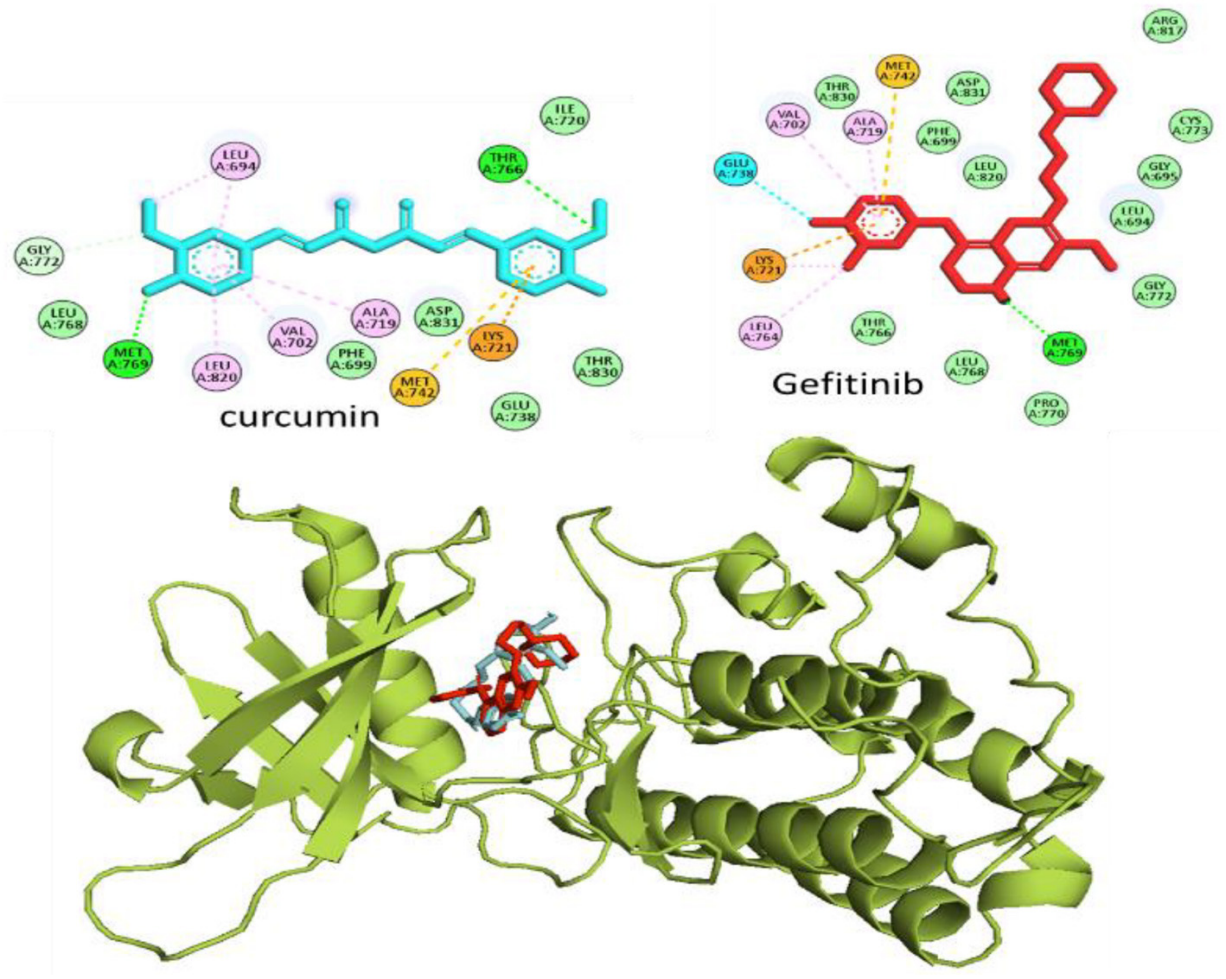
**Binding poses of CUR and positive control (Gefitinib) in the active site of EGFR, as well as the 2D interaction of these ligands with EGFR active site residues.** Abbreviations: CUR: Curcumin; EGFR: Epidermal growth factor receptor.

Docking studies were conducted to evaluate the potential effects of CUR on EGFR, using AutoDock 4.2. The prepared structures of EGFR, along with the ligands CUR and gefitinib (used as a positive control), were assessed. A grid box was centered at coordinates *X* ═ 23.24, *Y* ═ −0.4519, and *Z* ═ 56.12, encompassing the binding pocket. Both ligands were observed to interact within the same EGFR binding pocket and exhibited comparable binding affinities. Gefitinib achieved a binding affinity score of −7.9 kcal/mol, while CUR showed a slightly lower affinity at −7.5 kcal/mol. Figure 3 illustrates the binding poses of CUR (cyan) and gefitinib (red) within the EGFR catalytic pocket. Interaction analysis reveals that several common residues of EGFR interact with both ligands, as evidenced by the hydrogen bond interaction of MET769 with both CUR and gefitinib.

### MD simulation analysis

#### Root-mean-square deviation (RMSD) analysis

**Figure 4.**
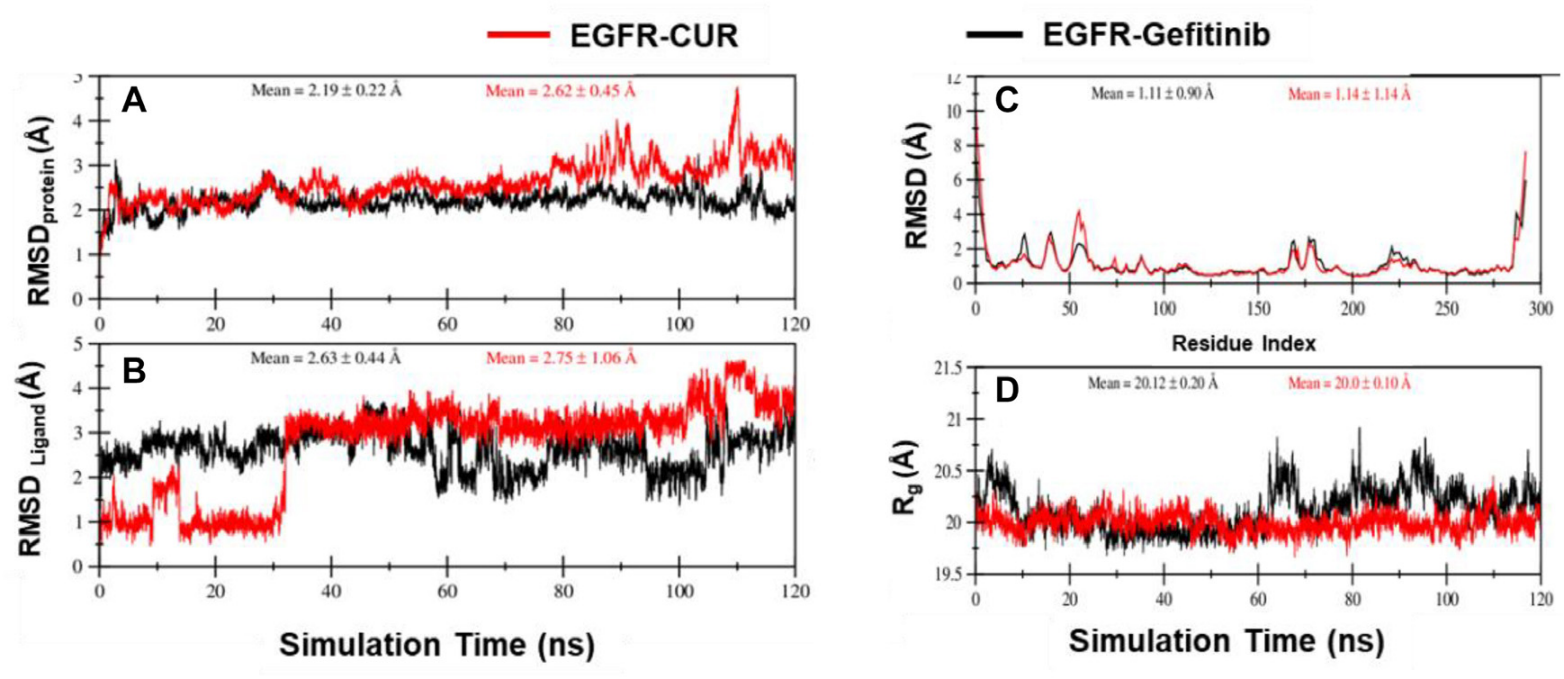
**The RMSD was calculated for the EGFR protein complexed with 01_Gefitinib (black line) and 02_CUR (red line) using the Bio3D module of R.** (A) Complexed with 01_Gefitinib and (B) 02_CUR; (C) Complexed with 01_Gefitinib and 02_CUR; (D) The RoG calculated for the EGFR protein complexed with 01_Gefitinib and 02_CUR. Abbreviations: CUR: Curcumin; EGFR: Epidermal growth factor receptor; RMSD: Root-mean-square deviation.

Both systems display similar RMSD values for the protein and ligand, indicating comparable deviations from their initial conformations over the 120 ns MD simulations. However, the EGFR–CUR system exhibits slightly higher RMSD values for both the protein and ligand, suggesting potentially greater conformational flexibility compared to the EGFR–Gefitinib system (Figure 4A and 4B).

#### Root-mean-square fluctuation (RMSF) and radius of gyration (Rg) analysis

Analysis of the RMSF profiles highlights differences in local structural fluctuations within the protein. Both systems show increased fluctuations at the C- and N-terminals, but the EGFR–CUR system displays slightly elevated RMSF values within specific residues (50–65) (Figure 4C and 4D). This suggests that the presence of CUR may induce localized conformational changes in certain regions of the protein.

#### Hydrogen bond analysis

**Figure 5.**
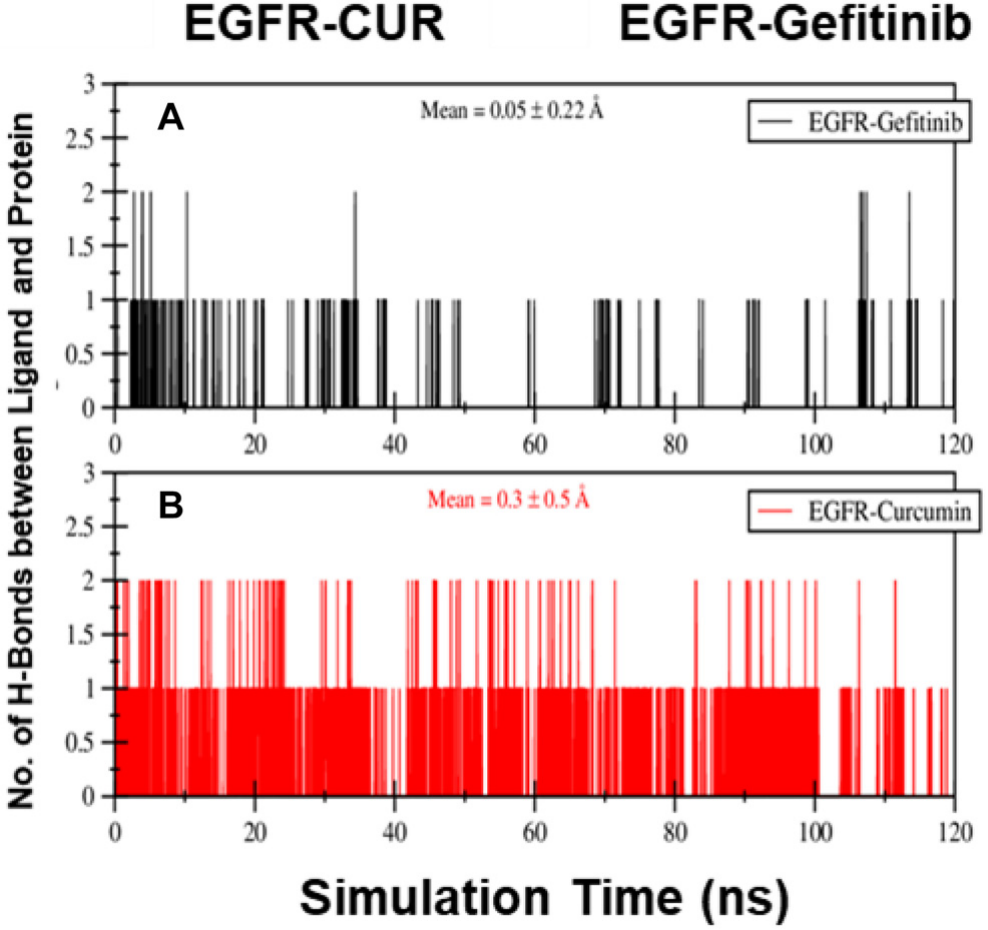
**Number of hydrogen bonds formed between the EGFR protein binding site and (A) 01_Gefitinib and (B) 02_CUR over a 120 ns simulation period.** Hydrogen bonds were calculated using the gmx_hbond command. Mean values and standard deviations are also displayed (A and B). Abbreviations: CUR: Curcumin; EGFR: Epidermal growth factor receptor.

The formation of hydrogen bonds between the protein and ligand is a key indicator of interaction strength. Notably, the EGFR–CUR system shows a higher average number of hydrogen bonds than the EGFR–Gefitinib system (Figure 5), suggesting that the interactions between the protein and CUR molecules are stronger and more extensive.

#### Center of mass (CoM) distance analysis

**Figure 6.**
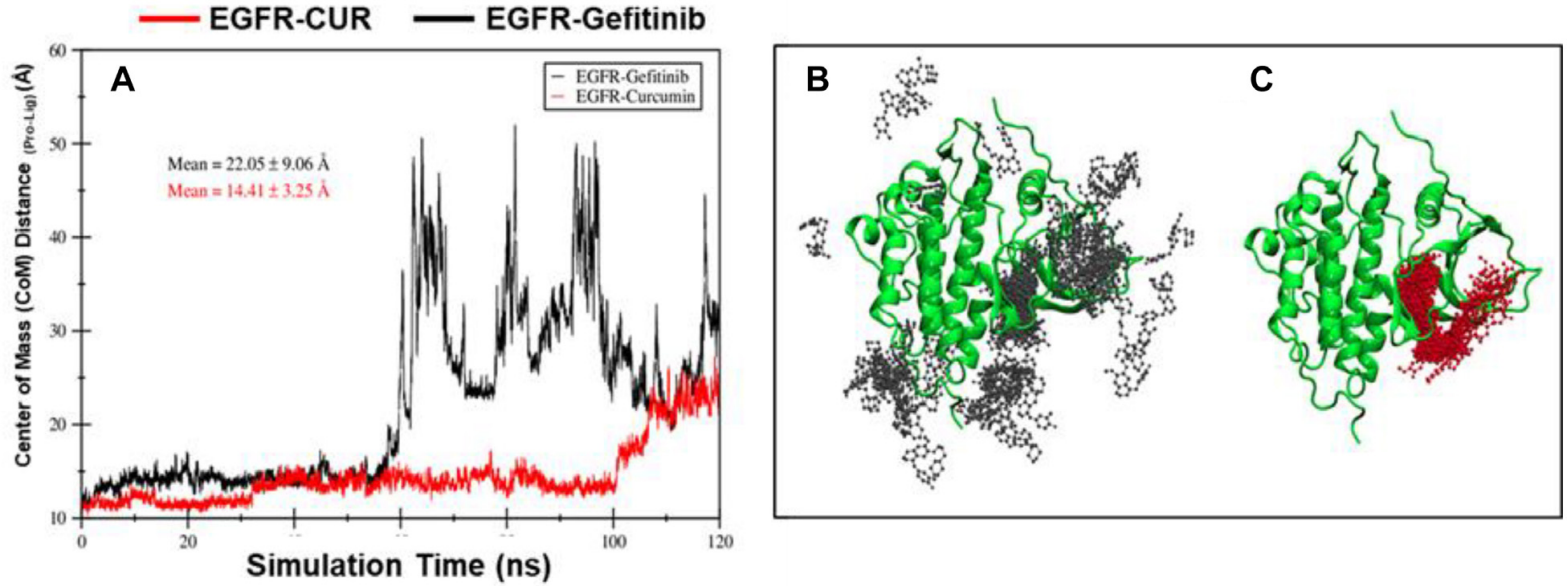
**During simulation time of 120 ns, panel (C) illustrates the distance fluctuation between the CoM of EGFR protein and the CoM of 01_Gefitinib (black line), and 02_Curcumin (red line) ligands (A).** The CoM distance was evaluated using the gmx_distance command. The mean and standard deviations are shown. In 01_EGFR_Gefitinib, the ligand left the binding site after first 48 ns. The 02_EGFR_Curcumin ligands remained bound until 100 ns and then slightly moved to a different site during 120 ns simulation time. Snapshots of 01_EGFR_Gefitinib (B), and 02_EGFR_Curcumin (C) complexes taken after every 1 ns of MD simulation (total 120 snapshots of ligands). In this snapshot, the protein is fixed at 0 ns. This indicates that 01_EGFR_Gefitinib ligands leaves the protein while 02_EGFR_Curcumin ligand remained in bound state during simulation time, however slight change in position is observed. This indicates that while the 01_EGFR_Gefitinib ligand detached from the protein, the 02_EGFR_Curcumin ligand stayed bound, with only minor positional changes. This observation is corroborated by the distance fluctuations shown in Panel (C). Abbreviations: CUR: Curcumin; EGFR: Epidermal growth factor receptor; CoM: Center of mass.

The CoM distance between the protein and ligand provides insight into their spatial arrangement. The EGFR–CUR system demonstrates a shorter mean CoM distance compared to the EGFR–Gefitinib system, indicating a tighter binding interaction between the protein and CUR (Figure 6). Additionally, in the EGFR–CUR system, the ligand remains within the binding pocket for a longer duration, further supporting the stability of the protein–ligand complex (Figure 6). In contrast, during the simulation, the EGFR–Gefitinib ligand detaches from the protein, as shown in the figure. However, the EGFR–CUR ligand remains bound, albeit with a slight positional shift. This observation is supported by fluctuations in the CoM distance between the EGFR protein and the EGFR–Gefitinib complex (black line).

####  MMGBSA calculations

**Figure 7.**
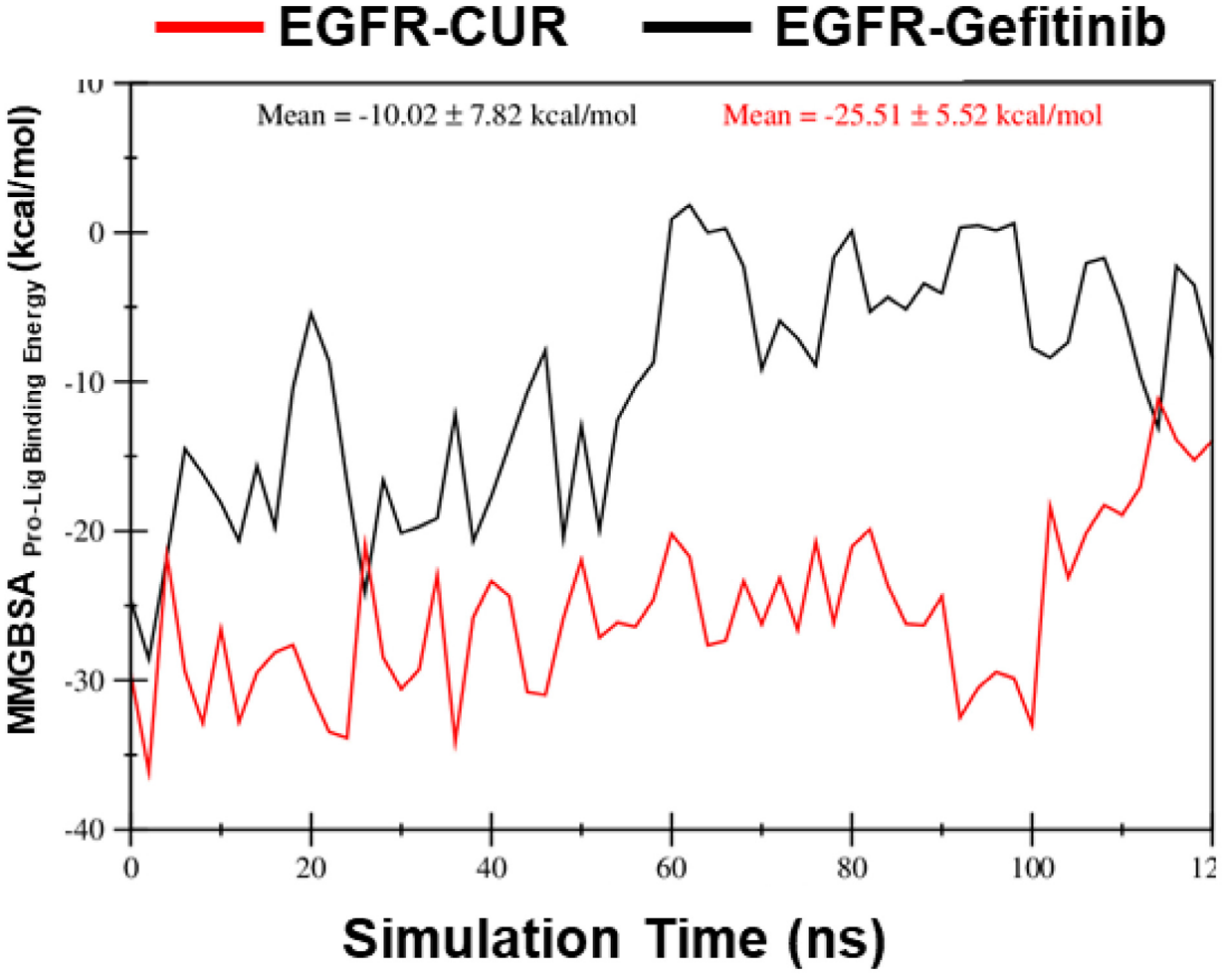
**The MMGBSA Gibbs free binding energies were calculated for snapshots taken every 1 ns of the EGFR protein complexed with 01_Gefitinib (black line) and 02_Curcumin (red line) ligands.** The results show that the 02_EGFR_Curcumin ligand binds more effectively to the protein compared to the 01_EGFR_Gefitinib ligand. Abbreviations: CUR: Curcumin; EGFR: Epidermal growth factor receptor; MMGBSA: Molecular mechanics generalized born and surface area.

Lastly, the MMGBSA binding energy calculations reveal significant differences between the two systems. The EGFR–CUR system exhibits a substantially more favorable mean MMGBSA binding energy than the EGFR–gefitinib system, indicating a stronger binding affinity between EGFR and CUR (Figure 7). This finding suggests that CUR may form more stable and energetically favorable interactions with the protein than gefitinib.

**Figure 8.**
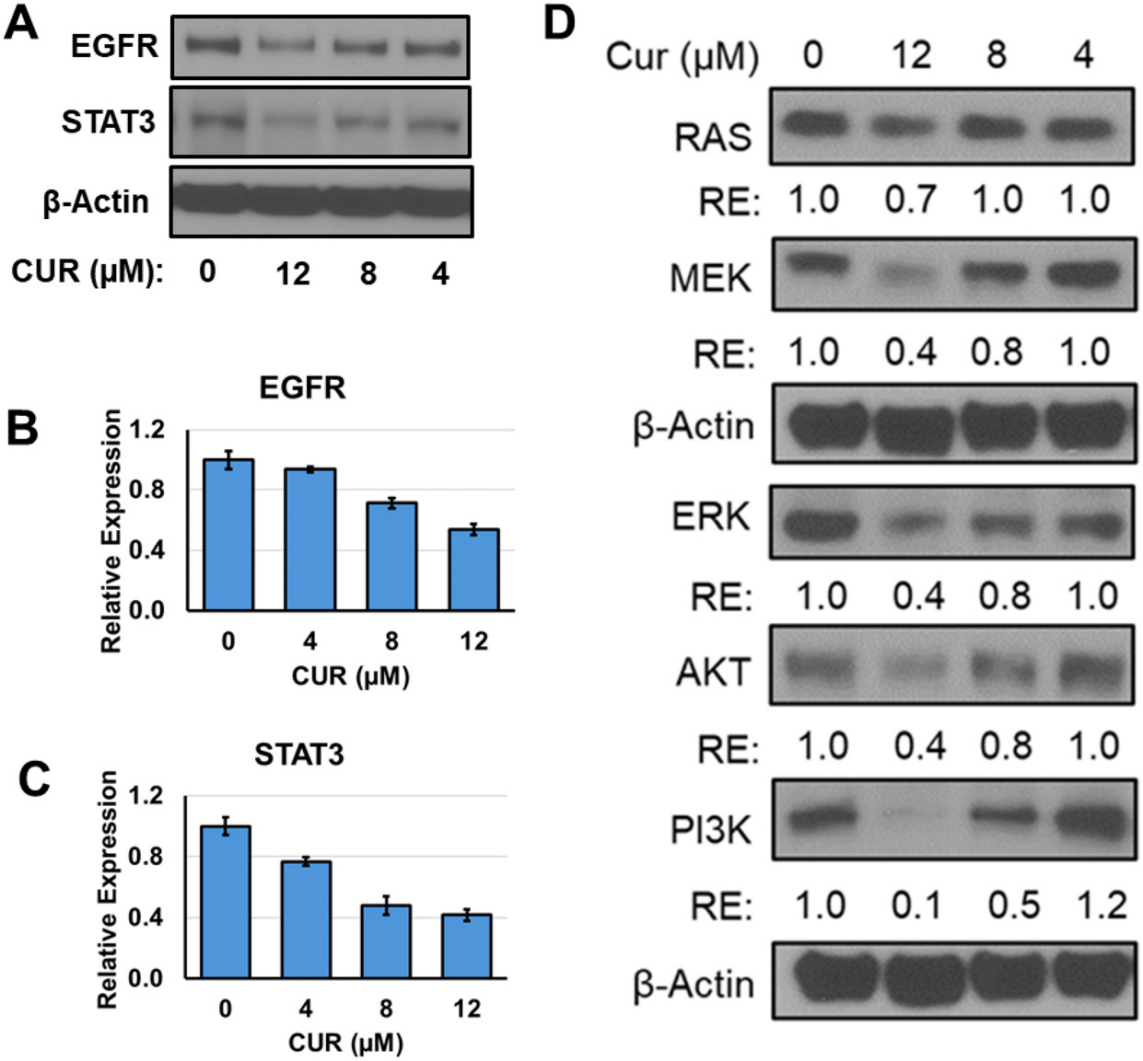
**Effect of curcumin on the EGFR and downstream signaling pathway in lung cancer cells. A549 cells were plated in six well plate and treated with different regimen.** After 48 h of treatments, cell lysates were prepared and probed for EGFR and other downstream signaling markers. Panel (A) shows the Western blot of EGFR and STAT3 expression. Panel (D) represents the Western blot images of effect of curcumin on EGFR downstream signaling markers and Panels (B–D) represent RE as analyzed by ImageJ software. Abbreviations: CUR: Curcumin; EGFR: Epidermal growth factor receptor; RE: Relative expression.

### CUR downregulated EGFR pathway in lung cancer cells

Promising results from in silico techniques, which indicated effective binding of CUR to the EGFR, prompted us to investigate the impact of CUR on EGFR signaling pathways. A549 lung cancer cells were treated with various concentrations of CUR. Cell lysates were then prepared, and Western blot analysis was performed to evaluate the levels of EGFR and several of its downstream targets: STAT3, RAS, MEK, ERK, AKT, and PI3K. Our results revealed that CUR treatment led to a dose-dependent downregulation of both EGFR and all downstream signaling markers, including STAT3, RAS, MEK, ERK, AKT, and PI3K (Figure 8). These findings suggest that CUR effectively inhibits EGFR signaling pathways involved in lung cancer progression.

## Discussion

Exosomes have significant potential for precise delivery of bioactive compounds, including small molecules, miRNAs, and other nucleic acid therapeutics [35–40]. The use of milk-derived exosomes for targeted delivery of small compounds has been previously documented [14]. Unlike cell-derived exosomes, which have relatively low yields, milk provides a plentiful supply of exosomes, facilitating the translation of discoveries to clinical settings. However, the availability of milk-producing species varies by region. For example, areas like the Middle East have large numbers of camels, which motivated us to explore camel milk as a source for exosome isolation. While bovine milk exosomes are well-established for biomedical applications and have undergone toxicity profiling, further studies on immunogenicity and detailed toxicity profiling are needed for camel milk-derived exosomes.

Despite progress, methods for isolating milk exosomes and combining them with bioactive molecules remain in the early stages of research and development. In our study, drug loading was performed using ethanol precipitation, followed by measurement of protein and curcuminoid concentrations with the BCA protein assay and UPLC, respectively. Our findings indicate that passive incubation is a straightforward and efficient method for loading drugs into exosomes. The concentration of individual curcuminoids was determined using standard curves for CUR. This formulation achieved a total curcuminoid load of around 20%. In recent studies, by optimizing parameters, such as centrifugation time for drug-loaded exosome collection and the drug-to-exosome ratio, we achieved a significantly higher drug load, reaching up to 60%.

CUR is a highly studied plant bioactive compound that shows potential therapeutic benefits against various ailments. CUR can selectively affect cancer cells with minimal toxicity toward normal cells [41]. However, its medicinal potential is limited by poor absorption into the bloodstream when administered orally [21, 42]. Despite numerous studies, CUR’s low water solubility continues to pose a significant obstacle to its absorption and therapeutic efficacy [21]. Various approaches have been explored to improve CUR’s dissolution and bioavailability, including encapsulation in synthetic vesicles, such as liposomes, polymeric nanoparticles, biodegradable microspheres, phospholipid mixtures, and hydrogels [43, 44]. Nonetheless, none of these delivery systems have advanced to clinical applications due to issues with toxicity, high cost, and/or lack of scalability [45]. Our current study demonstrates the capacity of camel milk-derived exosomes to effectively address the challenges associated with CUR’s bioavailability.

Exo-CUR appears to be an optimal nanoformulation due to its physical characteristics, including an average size of approximately 100 nm and a PDI of 0.26. An adequate drug load (∼20%) was achieved with the simple combination of CUR in the presence of a 10% ethanol concentration, which preserved the qualitative characteristics of the exosomes. This observation highlights the efficacy of the isolation procedure in producing well-defined exosomes suitable for therapeutic investigation. Furthermore, DLS analysis suggests that the isolated exosomes possess advantageous biophysical characteristics for therapeutic applications. These exosomes demonstrate promising attributes such as small size, a homogeneous population, and a moderately negative charge, all of which support their potential for therapeutic use. Although further research is needed to evaluate their precise effectiveness, this study establishes a solid foundation for leveraging exosome technology for medical applications. Additionally, we used Western blotting to assess the presence of exosomal markers CD63, CD81, and Alix in these exosomes, and we compared them with established bovine milk exosomes.

Lung cancer, particularly the drug-resistant A549 variant, poses a significant challenge in oncology. Our research aimed to investigate the therapeutic potential of Exo-CUR against both A549 and A549TR cells. The results were compelling: while exosomes and CUR alone displayed modest anti-proliferative effects, Exo-CUR exhibited significantly higher cytotoxicity. This enhancement suggests that exosomes act as efficient delivery vehicles, facilitating the entry of CUR into cells and potentially protecting it from degradation. The combination of exosomes and CUR presents a promising strategy for overcoming drug resistance in A549 lung cancer cells by leveraging improved delivery, multi-faceted anti-cancer activity, microenvironment modulation, and resistance mechanism evasion.

Furthermore, MD simulations and molecular docking methods were used to assess binding affinities to the EGFR receptor and the stability of both CUR and the control compound gefitinib. The results indicated that CUR possesses drug-like properties. Promising hydrogen bonding interactions were observed, demonstrating significant inhibitory potential against the EGFR receptor, with favorable binding energies. The binding affinities of CUR and gefitinib against the targeted protein were between −7.5 kcal/mol and −7.9 kcal/mol. Our findings suggest that CUR could be a potent compound for EGFR-associated lung cancer.

A comparison of the EGFR–Gefitinib and EGFR–CUR systems revealed differences in their structural dynamics and binding characteristics. While both systems showed similar overall structural stability, the EGFR–CUR system exhibited stronger interactions with the protein, as evidenced by higher hydrogen bond formation and more favorable MMGBSA binding energies. These advanced computational studies highlight CUR’s potential activity, though further wet lab experiments are necessary to validate these effects in vitro and in vivo. EGFR is a transmembrane family of receptors that abundantly expressed in human lung cancer cells. Upon activation by heparin-binding EGF-like growth factor (HB-EGF), the EGFR receptor undergoes heterodimerization and phosphorylation of its intracellular tyrosine kinase domain, triggering downstream signaling pathways [46--48]. Major EGFR-mediated signaling pathways that play key roles in lung tumor progression and metastasis include the RAS/MEK/ERK and phosphatidylinositol-3 kinase/Akt (PI3K/AKT) pathways [49, 50]. CUR treatment inhibited EGFR and downstream signaling markers in A549 cells in a dose-dependent manner, which may contribute to its anti-proliferative effects.

## Conclusion

In summary, this study demonstrated a biocompatible drug delivery system that utilizes camel milk-derived exosomes to deliver CUR for the treatment of lung adenocarcinoma. This nanoformulation addresses CUR’s low oral bioavailability, allowing for a reduced effective dose and enhanced efficacy. CUR-loaded exosomes significantly inhibited cell viability compared to free CUR and exosomes alone. Additionally, our study confirmed CUR’s potential as an EGFR inhibitor through various in silico and in vitro analyses. Therefore, camel milk-derived exosomes may offer an effective nano-delivery platform for CUR and other plant bioactives, many of which face similar bioavailability challenges. Despite the promising data, additional in vivo studies are needed to confirm the efficacy of this exosomal formulation.

## Supplemental data

## Data Availability

The data that support the findings of this study are available from the corresponding authors upon reasonable request.
